# A dual perspective on the microwave-assisted synthesis of HCN polymers towards the chemical evolution and design of functional materials

**DOI:** 10.1038/s41598-020-79112-5

**Published:** 2020-12-18

**Authors:** Lucía Hortal, Cristina Pérez-Fernández, José L. de la Fuente, Pilar Valles, Eva Mateo-Martí, Marta Ruiz-Bermejo

**Affiliations:** 1 Dpto. Evolución Molecular, Centro de Astrobiología (CSIC-INTA), Ctra. Torrejón-Ajalvir, km 4, 28850 Torrejón de Ardoz, Madrid Spain; 2 Instituto Nacional de Técnica Aeroespacial “Esteban Terradas” (INTA), Ctra. Torrejón-Ajalvir, km 4, 28850 Torrejón de Ardoz, Madrid Spain

**Keywords:** Molecular evolution, Astrobiology, Analytical chemistry, Nanoparticles, Structural properties, Synthesis and processing, Two-dimensional materials

## Abstract

In this paper, the first study on NH_4_CN polymerization induced by microwave radiation is described, where a singular kinetic behaviour, especially when this reaction is conducted in the absence of air, is found. As a result, a complex conjugated N-heterocyclic polymer system is obtained, whose properties are very different, and even improved according to morphological features, characterized by their X-ray diffraction patterns and scanning electron microscopy analysis, with respect to those produced under conventional thermal treatment. In addition, a wide variety of relevant bioorganics have been identified, such as amino acids, nucleobases, co-factors, etc., from the synthetized NH_4_CN polymers. These particular families of polymers are of high interest in the fields of astrobiology and prebiotic chemistry and, more recently, in the development of smart multifunctional materials. From an astrobiological perspective, microwave-driven syntheses may simulate hydrothermal environments, which are considered ideal niches for increasing organic molecular complexity, and eventually as scenarios for an origin of life. From an industrial point of view and for potential applications, a microwave irradiation process leads to a notable decrease in the reaction times, and tune the properties of these new series macromolecular systems. The characteristics found for these materials encourage the development of further systematic research on this alternative HCN polymerization.

## Introduction

HCN polymers are a heterogeneous family of very complex organic substances synthesized from net HCN, soluble salts of cyanide (NaCN, KCN or NH_4_CN), aminomalononitrile, diaminomaleonitrile (AMN, DAMN, the formal trimer and tetramer of HCN, respectively), or formamide under a wide range of experimental conditions. They are of great interest in studies about the origin of life and in the fields of prebiotic chemistry and astrobiology, since the first prebiotic synthesis of adenine by Oró in the early 1960s^1^. Moreover, these prebiotic polymers have recently inspired new coatings for potential biomedical and materials science applications^2–4^, such as bone contacting^5^, antibacterial coatings^6^ or passive filtration for stormwater/greywater disinfection^7^, and a new family of photocatalysers for the purification of wastewater^8^. On the other hand, the structural properties of these HCN polymers (and hence their potential applications) as well as the single biomolecules and related organics found in them via chromatographic analysis all depend directly on the synthetic conditions used for their production^9–13^. However, despite the considerable developments in the area of microwave-assisted organic synthesis and polymerization in recent decades^14–17^, only one report describes the production of HCN-derived polymers from formamide using microwave radiation^18^.

From the point of view of studies on the origin of life, astrobiology and prebiotic chemistry, microwave-assisted syntheses may simulate hydrothermal environments. Currently, these conditions are interesting because hydrothermal vents, either submarine^19,20^ or subaerial^21^, as well as nuclear geyser systems^22^, are suggested as preferential sites for increasing the molecular complexity that led to pristine biochemistry on early Earth. In addition, from a prebiotic perspective, aqueous HCN polymerization may be mainly considered as cyanide polymerization. This assumption can be acceptable because: (i) HCN polymerization is preferably conducted at alkaline pH 8–10^23^, (pK_a_ HCN = 9.2, 25 °C), and (ii) alkaline environments are considered to be ideal prebiotic niches for the accumulation of organics with biological interest^24,25^. From an industrial point of view and the potential applications of this singular polymeric system, the use of a microwave-induced polymerization should lead to, on one hand, a notable decrease in the reaction times; and on the other hand, important changes in their structural characteristics, since the temperature has a significant influence on the nature of these macromolecules^9^.

Thus, herein equimolar aqueous solutions of NaCN and NH_4_Cl (1 M) were heated at 180 °C under anoxic conditions (N_2_ atmosphere) using a microwave reactor as a first approach to study these innovative cyanide polymerizations. We mainly focus on anoxic conditions because a lack of air is necessary to simulate prebiotic environments. However, the data of additional experiments under microwave radiation conditions at 180 °C in the presence of air, and even those conducted under conventional heating, are discussed with comparative purposes to achieve a wide vision of the microwave radiation effect in NH_4_CN polymerization. A kinetic approach overview of the process was addressed by a gravimetric method^9,12^, and by means of UV–vis spectroscopy^26^. The insoluble NH_4_CN polymer collected (gel fraction) was characterised by Fourier transform infrared (FT-IR) spectroscopy, elemental analysis, X-ray diffraction (XRD), scanning electron microscopy (SEM), and gas chromatography-mass spectrometry (GC–MS). The aqueous soluble oligomeric/polymeric phase (sol fraction) was examined by UV–vis spectroscopic measurements and GC–MS and later concentrated using ultracentrifugation devices. All of these results are discussed considering the dual interest of these polymerizations in prebiotic chemistry as well as in materials science, providing new insights in both fields and encouraging the development of systematic research on microwave-initiated HCN polymerization.

## Materials and methods

### Aqueous NH_4_CN polymerization driven by microwaves

NH_4_CN polymerizations were conducted in water using equimolar amounts of KCN and NH_4_Cl (1 M, 11 mL), with a Biotage Initiator^+^ microwave reactor purchased by Biotage (Sweden), and 20 mL capacity vials. Both reagents, KCN and NH_4_Cl, were supplied by Panreac. The polymerizations were made either in the presence of air or under anoxic conditions at 180 °C and different reaction times were considered. The power supplied of the reactor is in the range from 0 to 400 W from magnetron at 2.45 GHz, with a constant power about 70 W for the temperature chosen in this work. The heating ramp for all reactions was set as 60 °C (30 s), 80 °C (10 s), 90 °C (10 s), and from this temperature up to 180 °C with a heating rate of 0.5 °C /s, followed by the programmed reaction time. This was done to avoid pressure peaks due to the likely build-up of ammonia inside the vial that would abort the programme. The maximum pressure reached was 15 bar. The final suspensions were vacuum filtered using glass fibre filters (Merck Millipore Ltd). Both the gel and sol fractions were collected and freeze-dried until a constant weight was reached. The pH values of all the experiments were measured using a pH Meter BasiC20 and electrode model 52.10. Proper cautionary measures were taken during the process (sensitivity of 97%). Polymer insoluble conversions, *α*, were calculated as *α* = [(final weight of insoluble NH_4_CN polymer/initial weight of CN^−^) · 100]^12^.

### Characterisation of the sol and gel fractions

For the structural characterisation by elemental analysis, FT-IR spectroscopy and powder XRD were performed using the same measuring equipment and parameters reported previously in reference^10^. The parameter records of the UV–vis spectra are described in references^9^ and ^26^. The use of centrifugal devices with different cut-offs for the concentration via ultrafiltration of the gel fractions resulting in subfractions with different apparent molecular weight ranges is described in ^27^.

### SEM

The surface morphology of the samples was performed by a ThermoScientific Apreo C-LV field emission electron microscope (FE-SEM) equipped with an Aztec Oxford energy-dispersive X ray microanalysis system (EDX). The samples were coated with 4 nm of chromium using a sputtering Leica EM ACE 600. The images were obtained at 10 kV.

### GC–MS

Prior to the injection on the GC–MS equipment, approximately 2 mg of the samples were: (1) hydrolysed: (a) using acid conditions heating at 110 °C in 6 M HCl for 24 h; (b) using moderately basic conditions and heating at 140 °C in a phosphate buffer solution (0.01 M, pH 8) for three days; (c) using basic conditions and heating at 110 °C in 5 N NH_4_OH for 24 h. Then, the samples were freeze-dried. (2) Each hydrolysed sample in 100 μL of BSTFA with 1% TMCS (BSTFA = N,O-bis(trimethylsilyl)trifluoroacetamide, TMCS = trimethylchlorosilane, obtained from Thermo Scientific) was heated at 80 °C for 3 h to obtain the respective trimethylsilyl derivatives. The derivatized samples were analysed by GC–MS using the same GC oven programme described in^28^, method a, or described in^29^, method b. For the sample hydrolysed using the phosphate solution, the solvent delay was 20 min.

## Results

The results of this study have been divided into several well differentiated but interrelated parts, motivated by the heterogeneous nature of these precipitation polymerization reactions. The first part addresses the black insoluble products that are formed as the polymerization progresses. A second section focuses on the study of soluble fractions, and finally, a part closer to analytical chemistry attempts to identify all the molecular species that can be generated from both reaction products after a hydrolytic process.

### Gel fractions study

#### Kinetic approach

**Figure 1:**
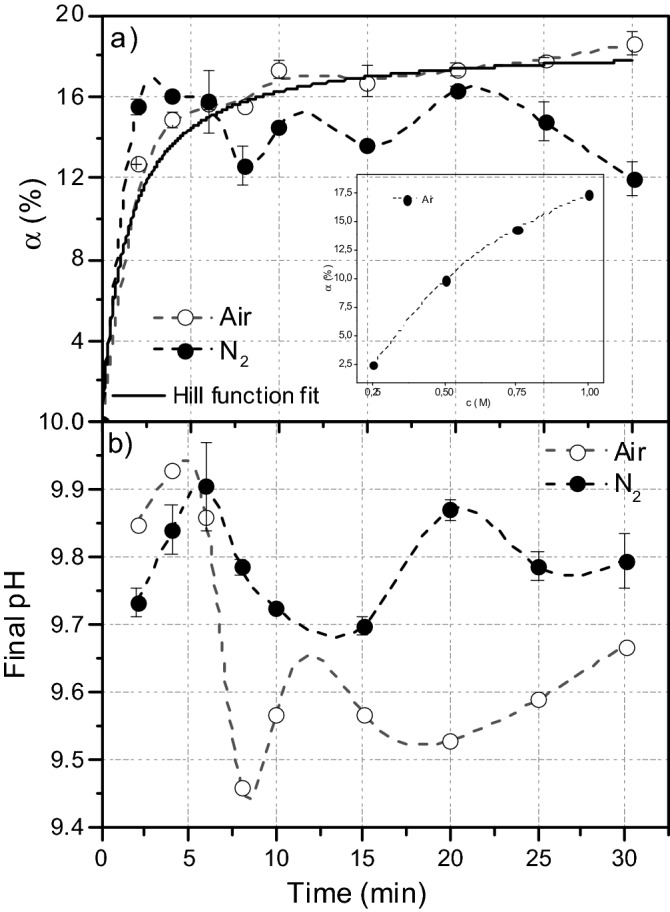
For microwave-assisted aqueous NH_4_CN polymerization (1 M), in the presence of air or under anoxic conditions: (**a**) Degree of conversion α *vs.* reaction time; (**b**) pH values of the suspensions corresponding to the crude reactions *vs.* reaction time. Note that the dashed lines are only to guide the eyes to more clearly see the evolution of the system against the reaction time. These lines are not fitted to any kinetic model; it is just a *slight connection* provided for the software programme. However, the solid line is fitted to a Hill equation of the data collected under air conditions. The inset plots represent the conversion α *vs.* NH_4_CN concentration for a fixed reaction time of 10 min for polymerizations conducted in the presence of air. At least three independent experiments were conducted to calculate the conversion data for the NH_4_CN polymerizations under anoxic conditions.

Two series of microwave-assisted NH_4_CN polymerizations, in the presence or absence of air, were conducted. For both series, polymerization products were separated into a continuous phase or sol fraction, soluble, and a dispersed phase or gel fraction, not water-soluble. Conversion degrees were evaluated from the insoluble fractions through gravimetric measurements^9,12^. The kinetic behaviour was studied and compared for both series (Fig. 1a). The nature of the technique renders it impossible to acquire conversion values for reaction times less than 2 min due to the necessary thermal profile used to reach the desired temperature of 180 °C, chosen as a first approach to understand the assisted-microwave cyanide polymerization chemistry. Significantly, for reactions in the presence of air, moderate conversions of ~ 12% appear at the lowest reaction times, 2 min, and almost immediately a conversion plateau of approximately 18% is reached. This value is lower than those obtained when the polymerization reactions were used under conventional heating (maximum yield ~ 40%), but it is in agreement with the fact that the conversion limit for insoluble cyanide polymers decreases with an increasing reaction temperature^9^.

Under air conditions, the system follows a kinetic profile previously observed when a classical method is used (from 38 to 90 °C); thus, a fitting to a phenomenological model based on a Hill equation can be properly made (*R*^*2*^ = 0.9993) (Fig. 1a). Considering the specification of the microwave reactor manufacturing, an assisted-microwave NH_4_CN polymerization at 180 °C for 30 min (the maximum time here applied) is equivalent to an analogous polymerization during 380 h (approximately 16 days) at 80 °C using a traditional thermal system. Taking into account that the conversion limit is reached at approximately 24 h at 80 °C, these data are in good agreement with previously reported results^9,12^. Moreover, when the polymerization time is fixed (10 min) and the initial concentration is increased from 0.25 M to 1 M (inset in Fig. 1a), the conversion values are also increasing, as was expected^10^. Therefore, under microwave radiation conditions in the range of the times considered here, the kinetic tendency of NH_4_CN polymerization qualitatively resembles classical polymerizations when air is present in the reaction environment.

In contrast, experiments performed under anoxic conditions presented a starkly and unexpected behaviour. In this particular case, one can see that at the lower reaction times (from 2 to 6 min), the conversion values are higher than those obtained under air conditions, as was expected. Higher values of conversion for insoluble NH_4_CN polymers were found at 75 and 90 °C when the polymerization reactions were conducted in the absence of air^12^. However, after an initial time of 6 min, the conversions began to present a surprising fluctuating behaviour, which is significant when taking the values of the standard deviations of these experimental data into account. The conversion for a time of 30 min is lower than that for 2 min. This oscillating kinetic reaction was never observed in any previous case described for NH_4_CN polymerizations. Its fit to a kinetic model is outside the scope of the present paper, but in the next sections, an attempt to relate this behaviour with that found for the sol fractions will be discussed. Finally, the pH of the reaction media was measured, taking into account that a correlation between the pH values of the crude suspensions and the biomolecular diversity of the corresponding hydrolysed acid was established^28^; this correlation is analysed later. In general, practically all the final pH values are higher than the initial pH of the reaction at 9.5. The pH data under anoxic conditions are higher than those in air, with the exception of the lowest polymerization time (Fig. 1b). Furthermore, the pH variation in the inert atmosphere shows a clear oscillating behaviour, with values in the range 9.7–9.9. However, this tendency is not as evident when the polymerization is conducted in the presence of air. In this case, after an initial stage where the pH reaches its maximum value, a sharp decrease is observed, pH < 9.5, and then the pH softly increases with the reaction time. On the other hand, the pH of the crude suspensions obtained from analogous standard NH_4_CN polymerizations increases with the reaction time (Fig. S1). In addition, when AMN is used as a precursor of HCN-derived polymers, the pH values of the final suspensions decrease with the reaction time^11^. Therefore, the assisted-microwave NH_4_CN polymerization under anoxic conditions seems to be a much more complex process than those polymerizations performed through a classical procedure.

#### Structural characterisation

Despite the dissimilar behaviour found in the previous section, the elemental analysis data of the corresponding insoluble NH_4_CN polymers do not show this same tendency (Fig. S2). For example, for the inert atmosphere series, the nitrogen percentage for gel fractions decreases slightly with the reaction time, while the carbon content increases in the same fashion. On the other hand, the O composition is constant, approximately 14–15%, along the entire time range studied.

**Figure 2:**
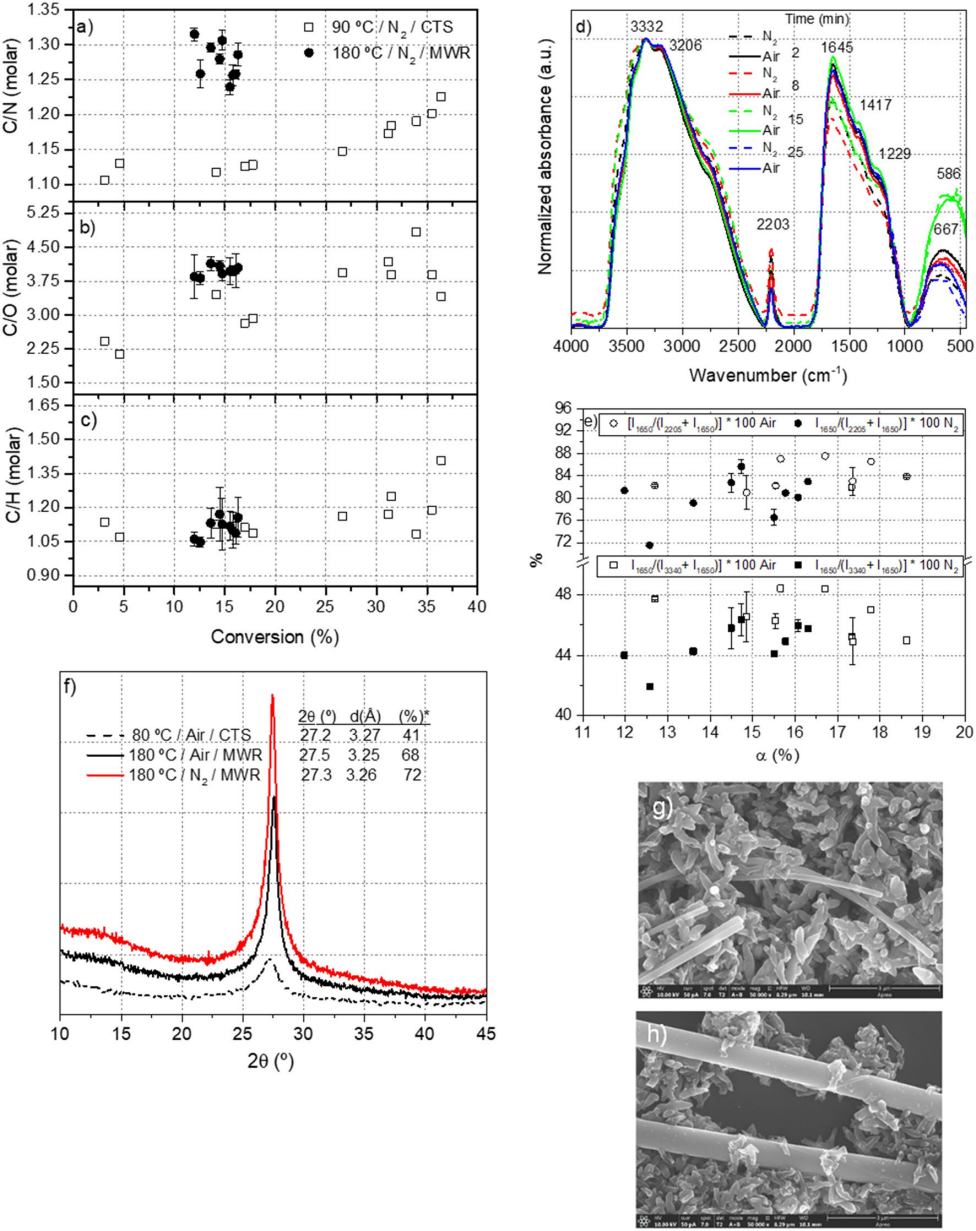
(**a**), (**b**) and (**c**) Molar relationships for insoluble NH_4_CN polymers, gel fractions, synthetized at 90 °C using a conventional thermal system (CTS) and at 180 °C via microwave radiation (MWR). Note that the values of C/H and C/N shown in Fig. 9 of Fernández et al. 2018^12^ correspond to relationships in weight; no molar relationships like those represented here. (**d**) Representative FT-IR spectra of insoluble NH_4_CN polymers (gel fractions) obtained using microwave-assisted syntheses at 180 °C. (**e**) Dependence of both the extension of reaction (*EOR*) and the extension of conjugation (*EOC*) relationships on the reaction time calculated from the FT-IR spectra. For each point represented, at least two spectra were recorded, which were obtained from independent samples. (**f**) XRD patterns of representative samples. The X-ray diffractogram of the sample synthetized at 80 °C using CTS is shown with a comparative propose (Ruiz-Bermejo et al. 2019^10^).* % of crystallinity of the sample. (**g**) and (**h**) SEM images of a representative sample.

In Fig. 2a–c, the different molar relationships as a function of the conversion are plotted for this series. For comparative reasons, the data for the corresponding NH_4_CN polymers obtained from a conventional method are also represented. Thus, a higher molar C/N ratio is found for the polymers produced under microwave radiation, as is clearly illustrated in Fig. 2a. This result is in agreement with previous works, in which high temperatures increase the deamination/denitrogenation processes^9^. The molar C/N relationship achieved a maximum value of 1.23 for NH_4_CN polymers synthetized at 90 °C and at a higher conversion under anoxic conditions^12^, but in the present case, this value is 1.31. In this way, it is confirmed and demonstrated that high temperatures lead to the production of insoluble NH_4_CN polymers with a lower nitrogen content than those synthetized at lower temperatures. (Fig. 2a). Unexpectedly, the samples characterised here present a molar C/O relationship ~ 4 (Fig. 2b), which is very similar to those ratios found for NH_4_CN polymerizations at high conversions conducted at 90 °C under anoxic conditions^12^. The content of oxygen in the macrostructure of the NH_4_CN polymers is independent of the presence of air in the reaction environment^12,30^, and instead depends on the temperature^9,12^. Moreover, the molar C/H relationships, as shown in Fig. 2c with values of approximately 1.10–1.15, conform to the values determined for the samples synthetized according to a classic procedure. In conclusion, anoxic microwave-assisted NH_4_CN polymerization produces macromolecular structures richer in carbon but less nitrogenized than analogues obtained using a conventional method.

Their FT-IR spectra present the same main features as those samples synthetized using a traditional methodology (Fig. 2d. For details about the assignment of these bands, please see^9,10,12,31^). The intensity of the nitrile bands (close to 2200 cm^−1^) is lower in reactions under the presence of air; conversely, the intensity of the bands related to unsaturated bonds (1645 cm^−1^, C=C and/or C=N) is lower in the anoxic series. Moreover, the width of the feature assignment to polar groups (approximately 3330 cm^−1^, such as amines, amides, and alcohols) is larger in the anoxic polymerizations. Quantitative data describing this behaviour can be acquired from the intensities between the higher peak at 1645 cm^−1^, *I*_*1645*_, and the nitrile band at 2200 cm^−1^, *I*_*2200*_. The following equation was used to determine the value of the extension of the reaction (*EOR*), *EOR* = [*I*_*1645*_/(*I*_*1645*_ + *I*_*2200*_*)*] · 100^12^. *EOR* values *vs.* conversion for both polymerization series are shown in the upper part of Fig. 2e. *EOR* values appear within a range of 76–87% and display no particular relationship with the conversion degree, though these data for polymerizations conducted in the presence of air are in a higher interval (from 81 to 87%) than those from anoxic polymerizations (from 76 to 85%, with a singular point at 71%). Significantly higher values can be found in a previous work, where it was demonstrated that this spectroscopic parameter for NH_4_CN polymerizations increases with the reaction temperature using traditional syntheses^9^. For example, the *EOR* values range for the insoluble NH_4_CN polymers synthetized at 90 °C is from 84 to 92%, increasing from the lowest value to the highest with the reaction time/conversion, as was expected^12^.

A comparative analysis of bands at 1645 and 3330 cm^−1^ results from the following equation to determine the extension of conjugation (*EOC*) in this macromolecular system, *EOC* = [*I*_*1645*_/(*I*_*1645*_ + *I*_*3330*_)]·100 (bottom part of Fig. 2e)^10^. Samples obtained in the presence of air present slightly higher *EOC* values than HCN polymers produced under anoxic conditions. It is also notable, as in the case of the *EOR*, that the *EOC* values are lower for samples synthesized by microwave radiation than those prepared with a classic method, in which the *EOC* is increasing with temperature^9,10^.

The XRD profiles (Fig. 2f) are very similar to those previously described for NH_4_CN polymers synthesized at lower temperatures^9,10^. An X-ray diffractogram of a sample synthetized under a classical method is incorporated in this figure to make the comparison easier, and crystallographic parameters are also indicated. The main peak at 2θ = 27.2°, usually assigned as the (002) feature of graphitic structures, is more intensive and significantly narrower in the NH_4_CN polymers synthesized here. This result agrees with the fact that the high temperature favours the production of more ordered macrostructures^9^, but also with the well-known pressure effect as a rearrangement factor. In addition, the grade of crystallinity of the samples synthetized under anoxic conditions is higher than those synthetized in the presence of air. As discussed in previous works^9,10^, these XRD profiles present extraordinary similarities with those observed for layered graphitic carbon nitrides g-C_3_N_4_^32^. As a result, the anoxic microwave radiation conditions yield highly rearranged NH_4_CN polymers, with a minor percentage of amorphous structures and an increase in ordered graphitic-like 2D polymer networks.

**Scheme 1:**
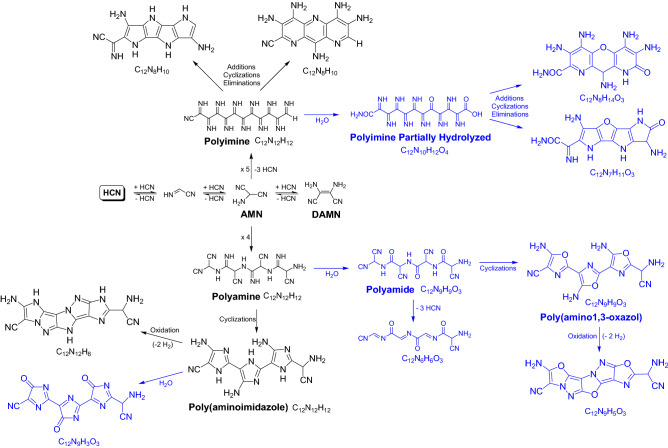
Pathways for the formation of NH_4_CN polymers from AMN according to recent studies in the literature^10,11^.

These characterization techniques indicate a complex nature for the polymeric system under study. Scheme 1 shows a broad summary for the aqueous polymerization of HCN, based on the previously reported hypothetical pathways for the synthesis of these polymers. Recent studies coincide in pointing to AMN as the main monomer that give rise to the growth of the polymeric chains. Two main polymerization routes can be seen, in addition to side reactions of hydrolysis, oxidation, etc.; and a wide variety of five- and six-membered nitrogen heterocycles are postulated, as it is described in detailed in the reference^10,11^.

In addition, it is also known that the morphology and textural properties of HCN-derived polymers are influenced by the synthetic conditions^2,10^. The spontaneous aqueous polymerization of AMN leads to the formation of based-coating films which final morphology depend on the coated material and the deposition time ^2^. On the other hand, the polymerizations in water of NH_4_CN and DAMN at 80 °C produced insoluble black polymers formed by spherical and oval particles or stacked sheets, respectively^10^. In the present case, long nanofibres are observed in representative samples of NH_4_CN polymers (Fig. 2g–h). Apparently, the number of these nanofibres is greater in the samples synthetized under anoxic conditions. These types of nanofibres were not previously observed. Other types of nanofibres were identified in HCN-derived polymers. In coated silica produced from the spontaneous aqueous polymerization of AMN^11^, and from the pyrolysis of formamide at 200 °C^8^, which were not so longer. Together with these nanofibres, a set of nanoparticle rice shapes were also observed (Fig. S3), which are very different in contour and size with respect to the analogous NH_4_CN polymers synthetized under a conventional method^2,10^.

Clearly, the synthetic conditions tune the properties of the NH_4_CN polymers, and the use of microwave irradiation, where high temperatures are reached quasi-instantly, results in materials with characteristics that have never been achieved until now and can increase the applicability of this particular family of polymers.

### Sol fraction study

The conclusions drawn above give rise to an approach for the study of the sol fraction of these precipitation polymerizations. To do this, its fractionation in combination with UV–vis spectroscopic features can be a useful methodology to be confronted with the information provided from the gel fraction. It is important to note that as has been proposed, soluble NH_4_CN oligomers/polymers are the precursors of black insoluble polymers due to an increase in the cross-linking reactions.

**Figure 3:**
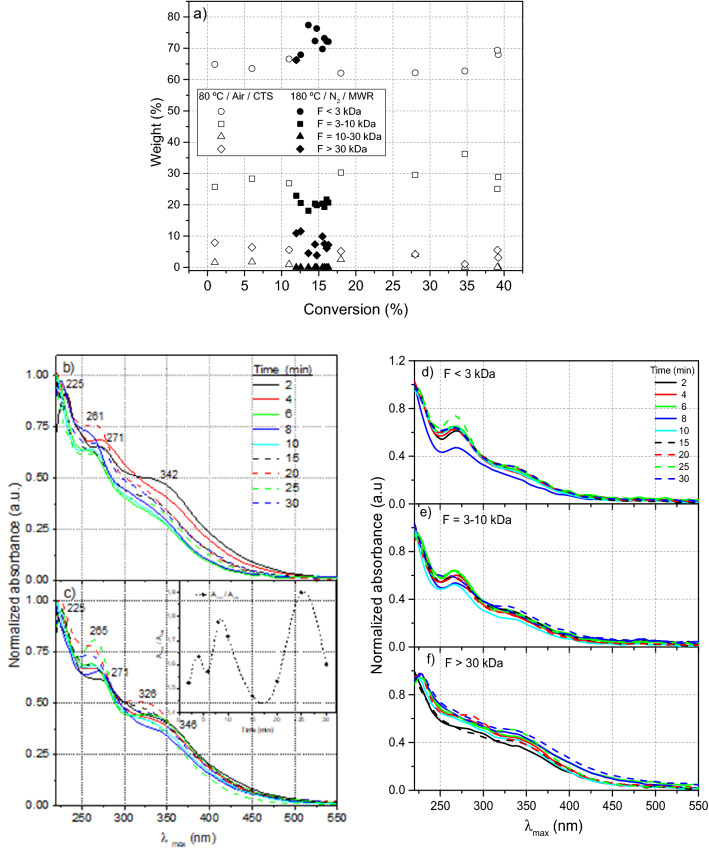
Soluble NH_4_CN polymers (sol fractions) were obtained using microwave-assisted syntheses at 180 °C. (**a**) Weight of sol subfractions concentrated by ultrafiltration using several centrifugal devices with different cut offs. In the picture the apparent molecular weights that present the different subfractions are indicated. The data of the polymers produced at 80 °C using a conventional thermal system (CTS) have never been reported before, and they are shown with comparative purposes. The percentage in weight of each subfraction was calculated as [(mg of sol subfraction) / (mg F > 30 kDa + mg F 10–30 kDa + mg F 3–10 kDa + F < 3 kDa)] · 100. Representative UV–vis spectra: (**b**) of raw sol fractions synthetized in the presence of air; (**c**) of raw sol fractions synthetized under anoxic conditions of a N_2_ atmosphere. The inset plot shows the evolution of the relationship between the main bands observed in these spectra over time; (**d**) of the lightest sol fractions with apparent molecular weights below 3 kDa; (**e**) of the light sol fractions with apparent molecular weights from 3 to 10 kDa; (**f**) for the heaviest sol fractions with apparent molecular weights above 30 kDa.

Considering the gravimetric methodology indicated in the first part of this work and the relationship between both phases, the relative ratios in weight of the soluble subfractions were represented against their corresponding conversion for their analogous gel fraction (Fig. 3a). The relative amount of the lightest subfractions (F < 3 kDa) is higher for the NH_4_CN polymerizations assisted by microwaves than those performed by means of a conventional method. In contrast, the percentage of the subfractions between 3 and 10 kDa is lower for the experimental conditions considered here. The subfractions between 10 and 30 kDa were not observed, and the amount of the heaviest fractions is comparable to classical conditions, except for the lowest conversions. The higher content in the lightest sol subfractions must be related to the lower conversion achieved for the gel fraction.

On the other hand, UV–vis spectra were registered for the whole sol fractions obtained in all conducted experiments (Fig. 3b,c) in order to further study the reaction kinetics as a complement to the first part of the present work. Three main absorption bands are observed at 225, ~ 260 and ~ 340 nm. The first peak at 225 nm is very similar for both series, and can be tentatively assigned to α,β-unsaturated nitriles, as well as amides and lactams^26^. The band at ~ 260 nm, in a range from 259 to 265 nm for anoxic reactions, can be related to N-heterocycles, such as purines and pyrimidines, which present a well-known absorption at this frequency. The thirst band undergoes a hypsochromic shift from 348 to 320 nm with the reaction time for the anoxic series, and it is well defined in all the monitored time ranges (Fig. 3c), whereas in the polymerization reactions conducted in the presence of air, it disappears for the longer reaction times (Fig. 3b). This might be assigned to N-heterocyclic systems on the macromolecular chain with π-extended conjugation^33^, and its shifting to blue might be due to the loss of auxochromic groups, such as amines, which is in good agreement with the deamination processes along the NH_4_CN polymerizations, as was discussed above. In addition, this band is dominant in the UV–vis spectra of the heaviest sol subfractions, which is not observed in the lighter ones (compare the spectra of Fig. 3d–f). These heavy sol subfractions are considered as the previous step, which leads to the precipitation of the gel fractions^27^ and their UV–vis spectra are in accordance with this fact, since they are highly conjugated systems in accordance with the structural results indicated above.

The relationships between the absorbance of the band at ~ 260 and at ~ 340 nm along the reaction time were represented to follow the NH_4_CN polymerization processes, similar to that designed in references ^9^ and ^26^ using the intensity of the main bands (plot inset in Fig. 3c). An oscillating behaviour was again observed. Previously, the band at ~ 260 nm had only been observed for long reaction times and temperatures above 70 °C^9^. According to conventional conditions, the increase in the intensity of the band at ~ 260 nm is followed by a decrease in the absorption at ~ 340 nm^9^, similar to that detected here for polymerization in the presence of air (Fig. 3b). Thus, it seems that the nature of the band at ~ 340 nm might be directly related to the kinetic behaviour of NH_4_CN polymerization using microwave radiation based on: (i) In the presence of air, this band nearly disappears, and the conversion *vs.* time curve reaches a “plateau”; (ii) Under anoxic conditions it is always present, undergoing a shift to the blue zone of the spectrum, varying in intensity, and the conversion *vs.* time curve also shows an oscillating behaviour. In addition, the UV–vis spectra of the subfractions of the NH_4_CN polymers synthetized using a conventional method (Fig. S4) presented different profiles than those obtained here under anoxic conditions.

These first sections have proven that microwave radiation has a notable influence on NH_4_CN polymerization, affecting the reaction pathway and extending the kinetics of the process, and therefore the properties of the final products achieved.

### Identification of polar molecules in the gel and sol fractions by GC–MS

**Figure 4:**
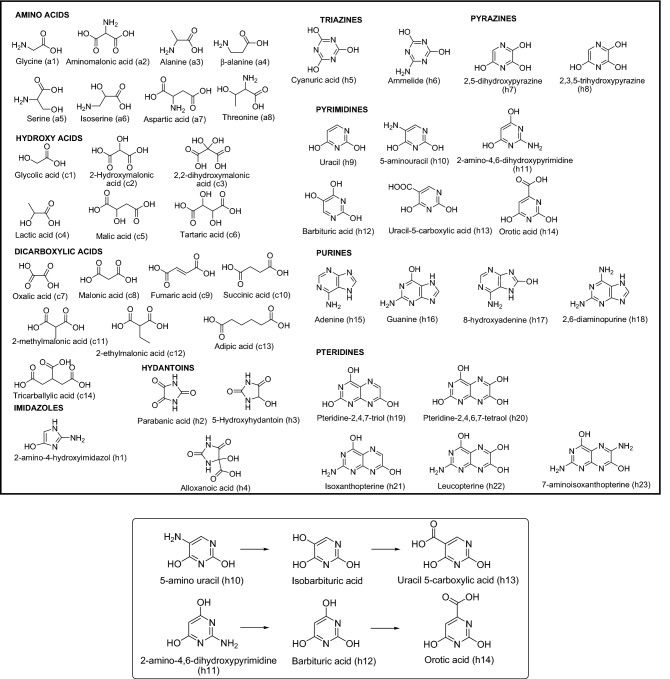
Upper square: All the polar organics identified by GC–MS from samples of NH_4_CN polymers synthetized under the microwave conditions present in this work. They are shown the tautomer form identified by GC–MS of each analyte as their TMS derivatives. Bottom square: Hypothetical oxidation reactions during the acid hydrolysis conditions used.

**Figure 5:**
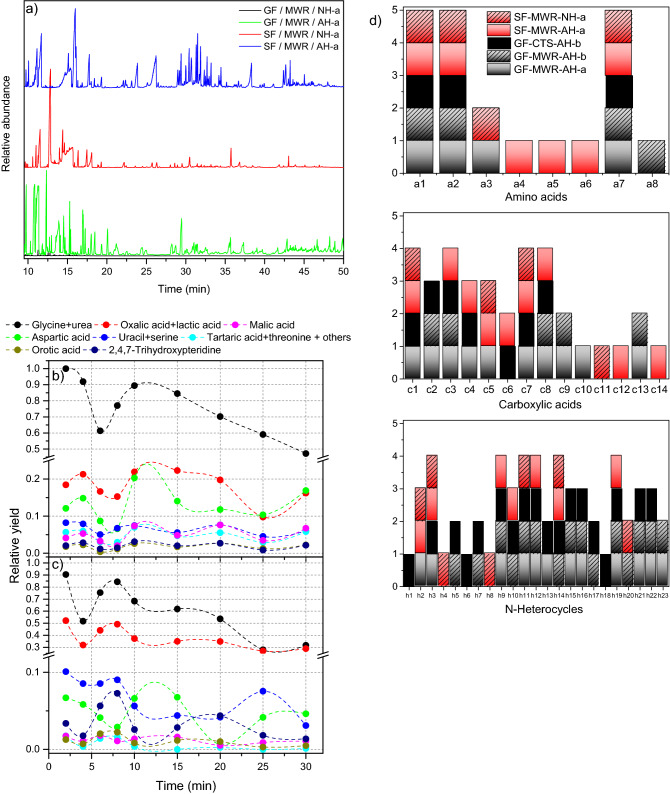
SF = sol fraction; GF = gel fraction; MWR = microwave radiation; CTS = conventional thermal system; AH = acid hydrolysed sample; NH = unhydrolysed samples; a and b = GC–MS methods (please see experimental section). (**a**) GC–MS profiles of unhydrolysed and acid hydrolysed samples from the NH_4_CN polymerization under anoxic conditions and microwave radiation using a reaction time of 30 min. Note that in the GC–MS profile of an unhydrolysed gel fraction sample, no well-defined polar organic compounds were identified and the relative intensity of the chromatogram signal is very low; (**b**) Semiquantitative analyses of the acid hydrolysed sol fractions. A value 1 is taken for the area of the urea peak plus the area of the glycine peak (3TMS) (please see the peak assignments in Figs. S5 and S6) in the sol fractions from the polymerization conducted at 2 min (Fig. S6). The other relative area values refer to that. Note that there are several saturated and coelution peaks. The assignment of the analytes was made using the retention time values and the profile of the mass spectra of the authentic standards. (**c**) Semiquantitative analyses of the acid hydrolysed gel fractions were performed in the same way as for the acid hydrolysed sol fractions. (**d**) Qualitative comparative analyses of the gel and sol fractions. The letter and number shown in the axis correspond to the analyte numeration indicated in Fig. 4.

The upper part of the Fig. 4 shows all the polar bioorganics and related organic compounds identified in this work via GC–MS. Additionally, GC–MS analyses of the representative samples from the anoxic polymerizations for the nine reaction times considered here were conducted for both the sol and gel fractions after acid hydrolysis (Figs. S6 and S7). The profiles of the chromatograms were the same for each of the two series considered, and only a relative variation in the analyte concentrations along the reaction time was observed. As an example, Fig. 5a shows the chromatograms of the acid hydrolysed sol and gel fractions obtained for a reaction time of 30 min (green line and blue line, respectively). No polar organics were identified in the corresponding unhydrolysed gel fraction (black line) and that the sol fraction GC–MS profile changes significantly with the hydrolysis process (red and blue lines). As expected, this unhydrolysed sol sample was saturated in urea and glycine and presented a significant amount of oxalic acid^34^.

Figure 5b,c shows the semiquantitative analyses of a selection of representative compounds found both in the gel and sol fractions. For the shortest reaction times, the samples are saturated in urea and glycine, and their concentrations decrease notably along the polymerization time in both series. However, the relative amounts of the other compounds fluctuate in a narrow range. Qualitatively, in the acid hydrolysed sol fractions, a greater diversity of amino acids was found, whereas a family of pteridines was better identified in the gel fractions (Fig. 5d). After acid hydrolysis, the gel fractions lead to a greater molecular diversity than the sol fractions, especially in relation to the number of N-heterocycles identified. The independent analyses of the sol and gel fractions instead of the bulk crude polymerizations were made in the way described only with practical analytical proposes. This previous separation obtained better and clearer analytical GC–MS results. However, in any case a full overview of all these analyses indicates that the NH_4_CN polymers synthetized under hydrothermal conditions are possible sources of important bioorganics, such as amino acids, carboxylic acids (some of them implicated in the r-TCA cycle, such as compounds **c5**, **c9** and **c10**) and several N-heterocycles.

Furthermore, the high temperatures increase the stability of the NH_4_CN gel fractions against acid hydrolysis conditions. For insoluble NH_4_CN polymers synthetized at 80 °C using conventional heating, only ~ 18% in weight is susceptible to acid hydrolysis^9^. However, this value is increased up to 50 ± 4% for the insoluble NH_4_CN polymers synthetized here under microwave radiation. The GC–MS profiles of the gel fractions after acid hydrolysis for both series (microwave radiation and conventional heating) are similar, but some significant differences were found. In a traditional method, the relative amounts of glycine, urea and oxalic acid remain constant along the course of the reaction (Fig. S8), and apparently a greater diversity in N-heterocycles is achieved (Fig. 5d). Nevertheless, when the same acid hydrolysed gel fractions from NH_4_CN polymerization assisted by microwaves are analysed using other GC–MS methods (variation in the temperature ramp of the oven), the peaks related to triazines, pyrimidines and purines are increasing (Fig. S9), revealing that the diversity in N-heterocycles from the insoluble NH_4_CN polymers produced under traditional conditions is comparable to the gel fractions synthetized here. The diversity in polar organics from gel fractions seems independent of the stability against hydrolysis.

**Figure 6:**
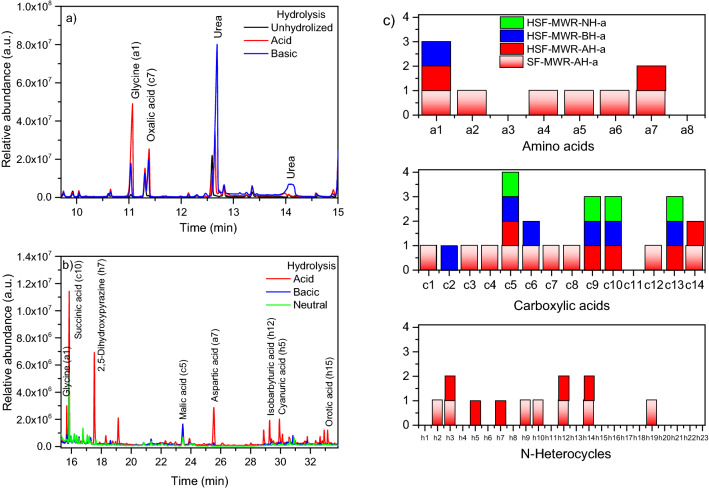
SF = sol fraction; HSF = heavy sol fraction (> 3 kDa); MWR = microwave radiation; AH = acid hydrolysed sample; BH = basic hydrolysed sample; NH = neutral hydrolysed samples; a = GC–MS method (please see the experimental section). (**a**) and (**b**) GC–MS profiles of a representative heavy sol fraction hydrolysed using several conditions. Note that the intensity of the peaks in (b) is much lower than that in (a). For this reason, the GC–MS chromatograms have been shown in two parts for a better view of the identified compounds. Only the analytes identified at a higher concentration are named. (**c**) Qualitative comparative analyses of several samples. The letter and number shown in the axis correspond to the analyte numeration indicated in Fig. 4.

Finally, for comparative purposes, the whole sol fraction synthetized using a reaction time of 30 min (the sample with the lowest amount of urea and glycine) was concentrated using centrifugal devices with a cut off of 3 kDa. The fraction above 3 kDa, free of salts, urea, glycine and oxalic acid, as shown in Fig. 6a, was analysed again using different hydrolysis conditions, since it is well known that these conditions have a significant influence on the qualitative and quantitative GC–MS analyses of the HCN polymers^35–37^. In addition, there are wide pH gradients in natural hydrothermal systems, which could contribute to the chemical evolution of HCN polymers in these types of environments. The basic hydrolysis results in a significantly greater amount of released urea (Fig. 6a), whereas the acid conditions yield a greater molecular diversity (Fig. 6b,c). The relative neutral hydrolysis conditions (pH = 8.5) only performed the identification of a few carboxylic acids in this soluble fraction (Fig. 6c). Note that the number of analytes identified is greater in the whole sol fraction than in its corresponding concentrated heavy fraction under the same acid hydrolysis conditions (Fig. 6c), indicating a greater molecular diversity of the light/oligomeric sol fraction with respect to its heavy fraction.

Therefore, in the present case no correlation was found between the final pH of the reaction (Fig. 1b) and the molecular diversity found in the corresponding NH_4_CN polymers (Fig. 5d). The molecular diversity was independent of the reaction time, and only variations in the yields of the analytes were observed. A larger number of polar analytes in the acid hydrolysed gel fractions is revealed despite the lack of identification in the corresponding unhydrolysed samples, and the lighter sol fractions are richer in polar organic compounds than their corresponding heavy fractions.

## Discussion

It was clearly demonstrated above that microwave radiation not only enhances the reaction rate, but also notably changes the conversion values and kinetics of the polymerization processes, leading to NH_4_CN polymers with novel properties. Therefore, taking the very complex chemistry of the HCN into account^38^, as well as the variations produced by the microwave radiation, the cyanide concentration is an experimental variable to study. Not only are concentrations lower than 1 M interesting from the point of view of the prebiotic chemistry, but also higher concentrations due to the potential applications in material science, where the experimental conditions must be optimised. Another important factor in hydrothermal systems is the temperature, with gradients from 2 to 350 °C in natural environments. Due to the technical restrictions of conventional microwave reactors, it is not possible to work above ~ 210 °C using water as the solvent. However, as it was shown, the temperature has a notable influence on the properties of the NH_4_CN polymers, and the synthesis assisted by microwaves allows us to approach a wide range of temperatures, which induces modifications in the textural and morphological features of these substances. In the present case, nanofibres were identified, which are interesting due their potential applications in several areas, such as tissue engineering, drug delivery, cancer diagnosis, lithium-air batteries, optical sensors, air filtration and sportswear textiles. Previously, it was suggested that HCN-derived polymeric nanoparticles could be incorporated as novel fillers to develop new polymer composites^10^. Here, for the first time, the identification of NH_4_CN polymeric nanoparticles was supported. It is true that in this work a mixture of nanofibres and nanoparticles is obtained, but a proper variation of the synthetic conditions might reach the production of pure morphology. The pressure parameter is directly related to the temperature in the microwave-assisted polymerizations, and high pressures seem to produce more ordered and crystalline NH_4_CN polymers than those synthetized under atmospheric pressure. They can be considered graphite-like 2D structures and seem to have a direct relationship with g-C_3_N_4_, which are semiconducting structures and can induce photoredox reactions, notably enriching the prebiotic reaction chemical space^39^. Thus, a systematic study jointly considering the temperature and pressure can help with understanding the influence of these factors as a whole in the structural properties of HCN-derived polymers and for extensions in the possible role of these substances in plausible prebiotic photochemical processes and for developing new 2D-multifunctional materials. Finally, HCN-derived polymers can be obtained from different starting reagents, as discussed in the introduction, and previous studies described that their properties depend on the monomers used^2,10^. Here, NH_4_CN was used as the reactive, but for the design and development of new multifunctional materials, other reactants can be considered.

On the other hand, the origin of life on Earth is one of the great puzzling problems of current science. The chemistry of HCN and, for extension, HCN-derived polymers, are considered to play key roles in the formation of the first and primeval protometabolic and informational systems, as well as in the hypothesis of the *“RNA-world”*^28,37,40–43^. Currently, alkaline environments are proposed as a good place to develop prebiotic HCN chemistry^21,40,44,45^, and the HCN could have an important role in the formation of complex organic molecules under hydrothermal environments^46–48^. Here, the NH_4_CN oligomers/polymers synthetized under simulated hydrothermal conditions are precursors of a rich variety of bioorganics and related compounds. Some of them have previously been reported in HCN polymerization experiments, and other ones were identified for the first time, such as ammelide (**h6**), the pyrazines 2,5-dihydroxypyrazine (**h7**), 2,3,5-trihydroxypyrazine (**h8**), the pyrimidines 2-amino-4,6-dihydroxypyrimidine (**h11**), uracil-5-carboxylic acid (**h13**) and tentatively the pteridine 7-aminoisoxanthopterine (**h23**) (Fig. 4). These results increase our knowledge about the molecular diversity derived from NH_4_CN oligomers/polymers. Beyond the nucleobases identified (uracil (**h9**), adenine (**h15**) and guanine (**h16**)), it is interesting to detect new triazines and pyrimidines, since recently cyanuric acid (**h5**), barbituric acid (**h12**), melamine and 2,4,6-triaminopyrimidine have been proposed as no canonical nucleobases in a *pre-RNA world* due to their capability to form ribonucleosides and supramolecular assemblies that are held by Watson–Crick type hydrogen-bonded base pairs. These heterocycles can be formed from cyanamide, its derivatives (malonic acid and urea) and AMN ^49,50^*.* All of these N-heterocycle precursors are known to be formed from HCN ^13^. The analytical GC–MS methods used in this study are not specific for the identification of N-heterocycles. For example, barbituric acid (**h12**) and isobarbituric acid (bottom part of the Fig. 4) present the same retention time and mass spectra using the chromatographic methods described here (based on analyses of authentic standards; data not shown), and different peaks correspond with several isomers of pyrimidines, and a significant number of coelution peaks were found (Figs. S6–S9). Additionally, some analytes identified are oxidation products of others that may be produced during the hydrolysis processes (bottom part of the Fig. 4). Therefore, the plausible key role of triazines and pyrimidines in a “*pre-RNA world”* encourages the development of specific analytical methods for their identification in the complex mixture formed under simulated hydrothermal conditions, considering the potential of these environments in the hypothesis about the origin of life and the better plausible conditions that might lead to an increase in molecular complexity. In addition, HCN-derived polymers present a growing interest in studies about the habitability of the icy satellites of the Solar System since the presence of HCN in the subsurface oceans of these freeze moons might also be assumed. These satellites present active hydrothermalism, and HCN might be produced in these extraterrestrial oceans, taking its easy formation under analogous terrestrial hydrothermal conditions into account^47^. The potential presence of HCN in these moons may lead to a complex organic chemistry^51,52^.

## Conclusions

Microwave radiation has a marked influence on NH_4_CN polymerization, especially when the reactions are conducted in the absence of air, which is categorically demonstrated, as its efficiency is closely linked to the experimental conditions, probably due to relevant mechanistic variations associated with a singular kinetic behaviour. This fact leads to polymers whose structural and textural features can be tuneable, mainly according to XRD patterns and SEM analysis, when an innovative polymerization method is applied to this robust prebiotic reaction. These NH_4_CN polymers have a rich organic chemistry and are precursors of several important bioorganics, such as amino acids, carboxylic acids, an elevated number of N-heterocycles, such as hydantoins, nucleobases, no canonical nucleobases of a possible *pre-RNA world,* and co-factors. In addition, their final properties indicate that they are suitable candidates for the development of new multifunctional materials. Thus, basic research in astrobiology inspires the development of novel materials and motivates the investigation of unexplored but promising HCN microwave-driven chemistry.

## Supplementary Information

Supplementary Figures.
