# Supplementary material for: A dual perspective on the microwave-assisted synthesis of HCN polymers towards the chemical evolution and design of functional materials
Source: Sci Rep. 2020 Dec 18;10:22350. doi: 10.1038/s41598-020-79112-5 (PMC7749158; doi:10.1038/s41598-020-79112-5)
Supplement: Supplementary file 1 — Supplementary Figures. [file 41598_2020_79112_MOESM1_ESM.pdf]

SUPLEMENTARY MATERIAL

A DUAL PERSPECTIVE ON THE MICROWAVE-ASSISTED SYNTHESIS OF HCN POLYMERS TOWARDS THE CHEMICAL EVOLUTION AND DESIGN OF FUNCTIONAL MATERIALS

Lucía Hortal, Cristina Pérez-Fernández, José L. de la Fuente, Pilar Vallés, Eva Mateo-Martí, Marta Ruiz-Bermejo

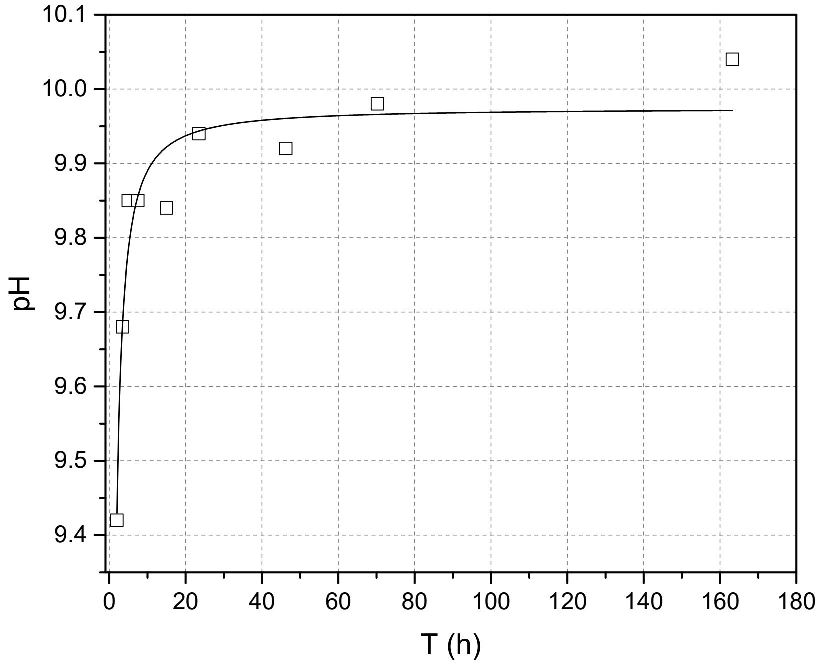

Figure S1. pH values of the suspensions corresponding to the crude reactions of the NH_4CN polymerizations at 75 ºC under nitrogen atmosphere (for details about these reactions see Fernández et al. 2018. Ref [12] in the main manuscript). The increasing of the pH value with the reaction time also can be fitted to a Hill equation, a sigmoidal growing, with a good fitting (R^2= 0.9359), as the data conversion *vs* reaction time for the corresponding insoluble NH_4CN polymers obtained under the same conditions.

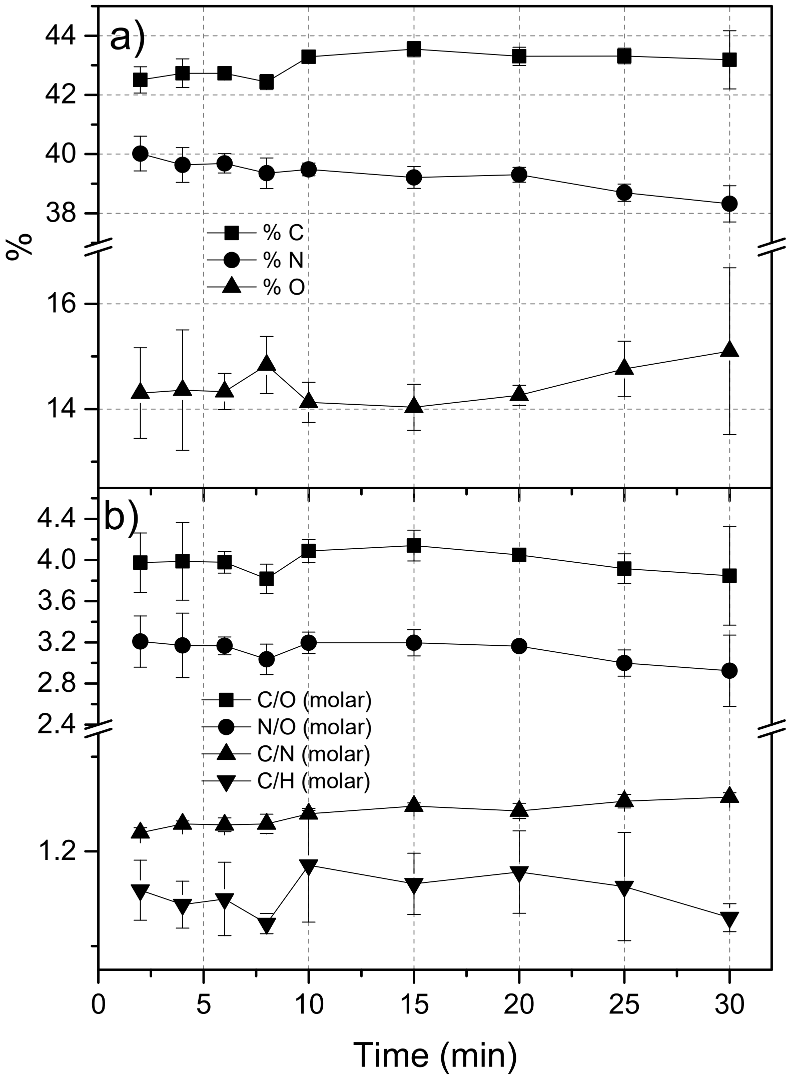

Figure S2. For insoluble NH_4CN polymers, gel fractions, synthetized at 180ºC using microwave radiation under anoxic conditions: a) Elemental analysis data; b) Molar relationships. In the x axis is indicated the reaction time for each polymerization.

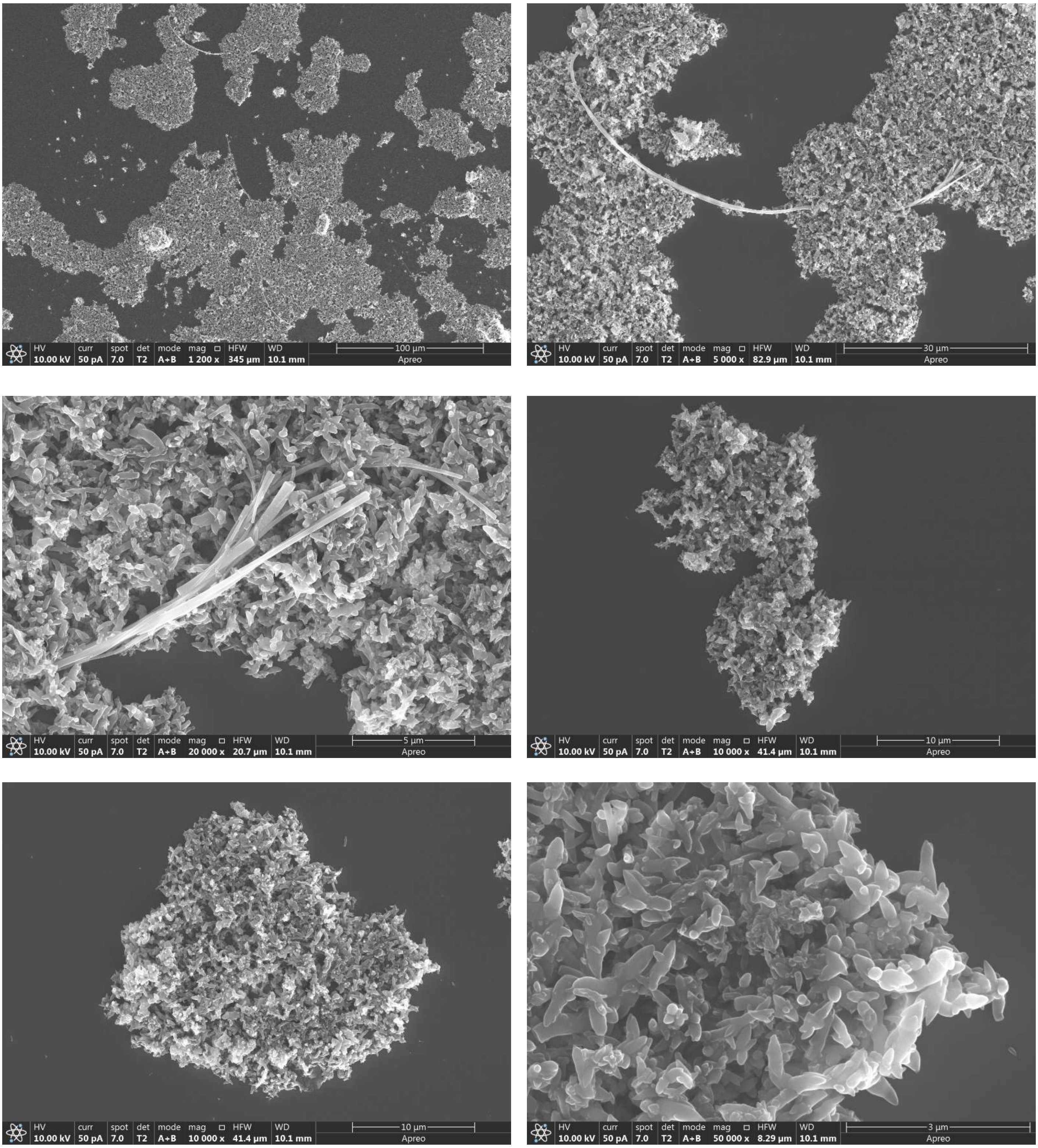

Figure S3. SEM images of NH_4CN polymers, gel fractions, synthetized under hydrothermal conditions at 180ºC in the presence of air.

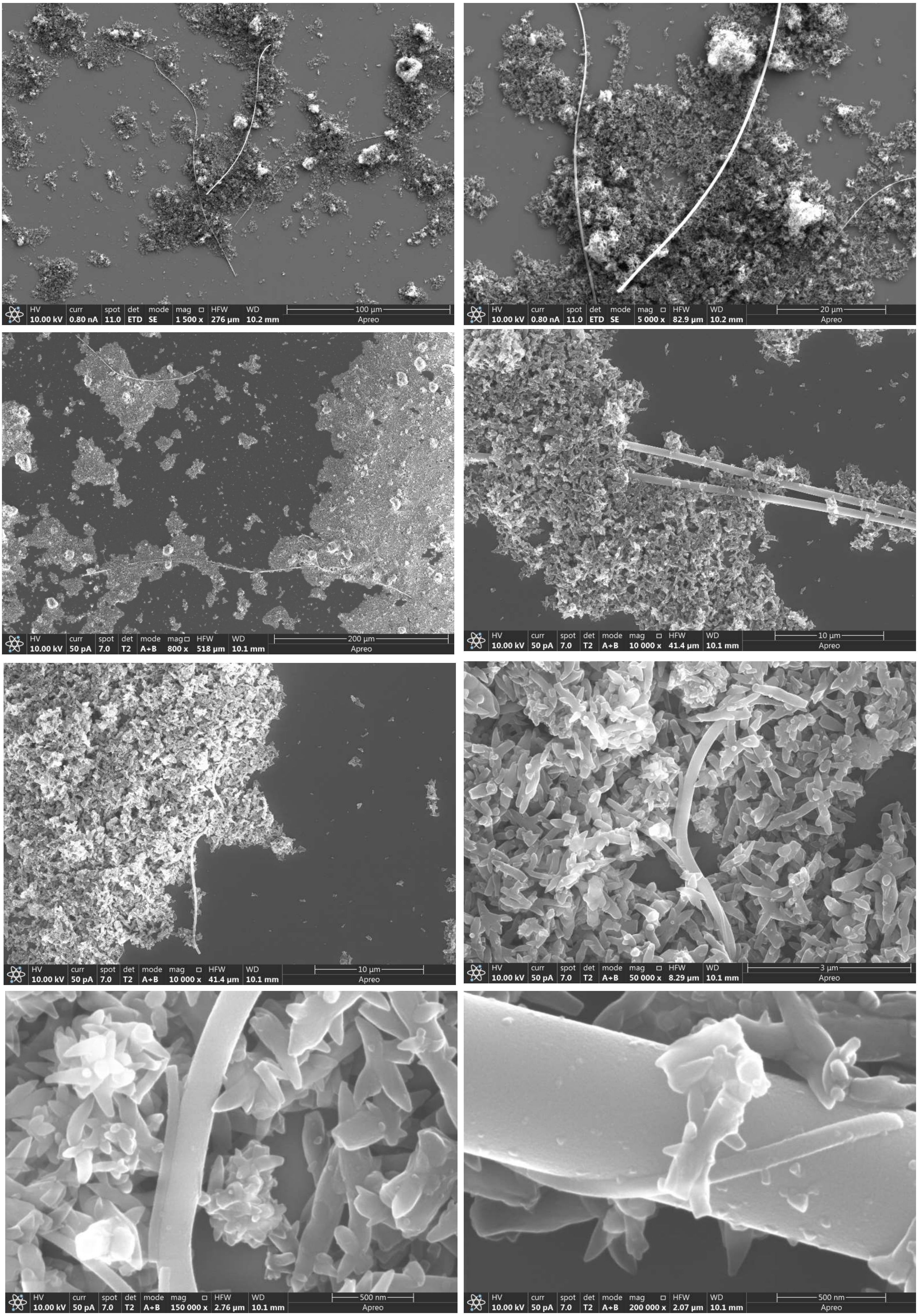

Figure S4. SEM images of NH_4CN polymers, gel fractions, synthetized under hydrothermal conditions at 180ºC under anoxic conditions.

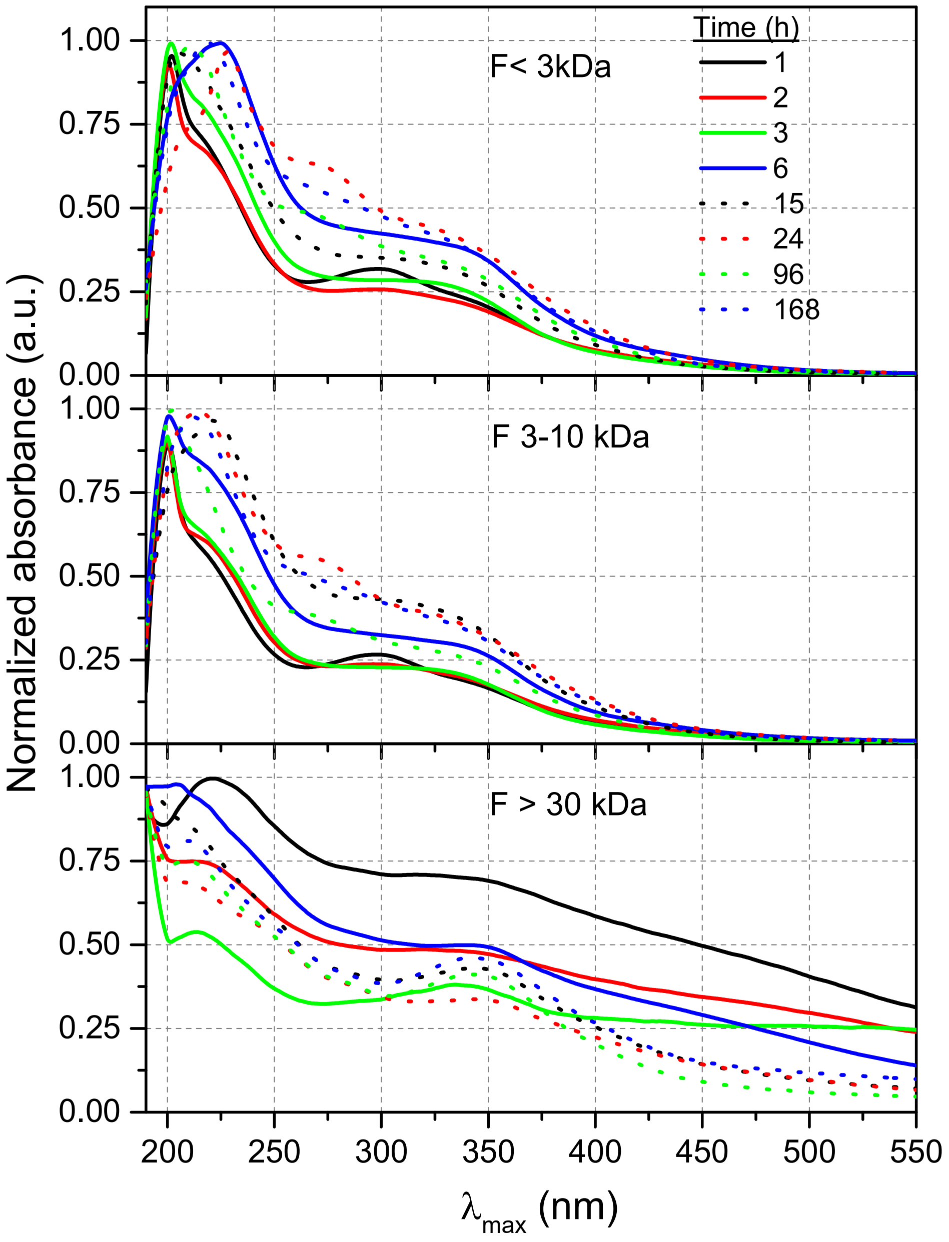

Figure S5. Representative UV-vis spectra of sol subfractions obtained after ultrafiltration from NH_4CN polymerizations carried out at 80ºC in the presence of air. The time of polymerization for each fraction is indicated in the graph.

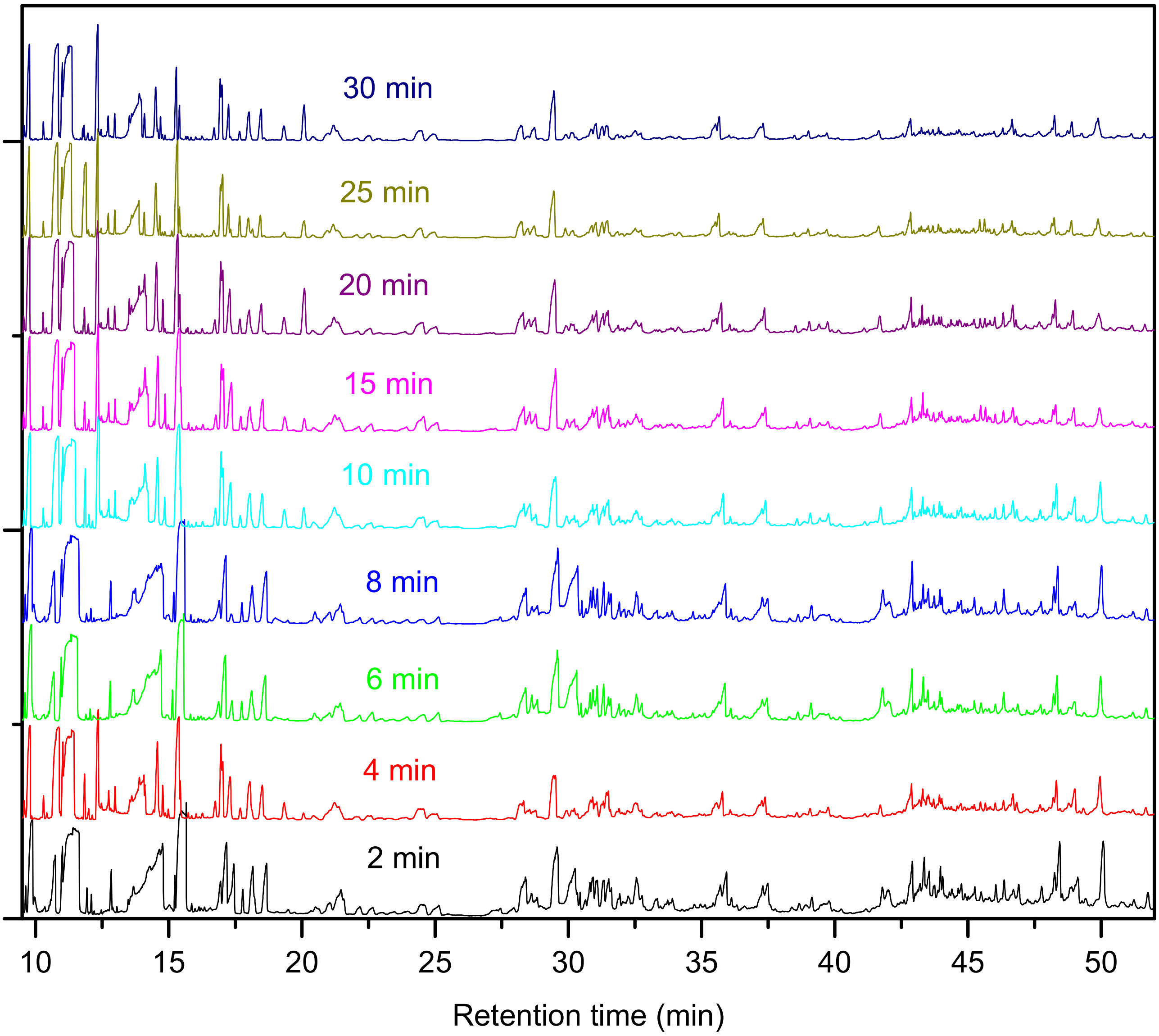

Figure S6. Representative GC-MS chromatograms of insoluble NH_4CN polymers, gel fractions, synthetized under hydrothermal conditions at 180ºC using a N_2 atmosphere, after acid hydrolysis. The time showed over chromatogram is the polymerization time. The following analytes were identified (the retention time is indicated): 9.41 min lactic acid, 9.77 min glycolic acid, 10.29 min alanine, 10.83 min glycine (2 TMS), 10.99 min oxalic acid, 12.34 min urea (3 TMS, co-elution peak), 12.73 min malonic acid, 15.33 min glycine (3TMS), 15.41 min succinic acid, 16.26 min fumaric acid (co-elution peak), 16.95 min uracil (co-elution peak), 17.25 min N-formylglycine, 18.0 min 2-hydroxymalonic acid, 19.35 min malonic acid, 20.97 min dihydroxymalonic acid, 21.33 min parabanic acid (co-elution peak), 22.54 min malic acid, 22.89 min adipic acid, 23.81 min 5-hydroxyhydantoin, 24.45 + 24.95 min aspartic acid, 27.85 min isomer of barbituric acid, 29.89 min aminomalonic acid, 30.72 adenine, 30.98 min + 31.04 min + 31.27 min isomers of 2-amino-4,6-dihydroxypyrimidine (NIST), 33.66 min cyanuric acid, 37.31 min uracil-5-carboxylic acid, 41.64 min 2,4,7-trihydroxypteridine, 43.69 guanine, 44.57 min isoxanthopterine, 46.31 min leucopterine, 48.16 min 7-amino-isoxanthopteine (tentatively assigned by its mass spectra). In this case the GC-MS method used was the method A described in [28].

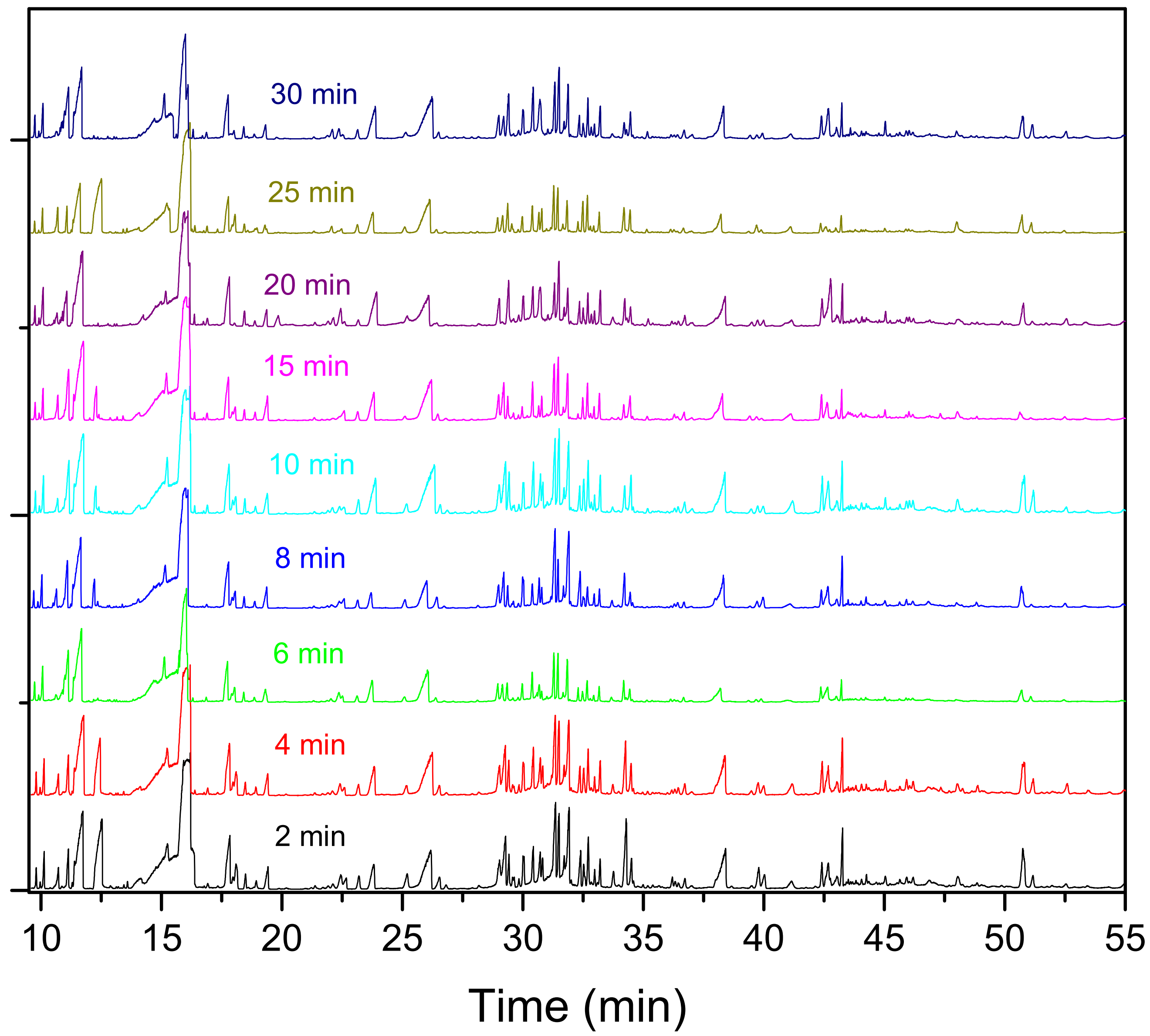

Figure S7. Representative GC-MS chromatograms of soluble NH_4CN polymers, sol fractions, synthetized under hydrothermal conditions at 180ºC using a N_2 atmosphere, after acid hydrolysis. The time showed over chromatogram is the polymerization time. The following analytes were identified (the retention time is indicated): 9.73 min lactic acid, 10.06 min glycolic acid, 11.07 min glycine (2 TMS), 11.55 min oxalic acid (co-elution peak), 13.17 min malonic acid, 13.60-15.33 min urea (saturated peak), 15.65-16.85 min glycine (saturated peak, 3TMS), 16.38 min 2-ethylmalonic acid, 16.75 + 17.77 min uracil (co-elution peaks, uracil + serine), 17.93 min isoserine, 18.05 min N-formylglycine, 19.86 min β-alanine, 21.88 min dihydroxymalonic acid, 22.31 min aminomalonic acid, 22.47 min parabanic acid, 23.76 min malic acid, 25.09 min 5-hydroxyhydantoin, 26.09 min aspartic acid, 29.37 min barbituric acid, 29.80 min 5-aminouracil, 30.98 min tartaric acid, 31.28 min threonine, 31.71 min isomers of 2-amino-4,6-dihydroxypyrimidine (NIST), 32.80 min tricarballylic acid, 33.80 min orotic acid, 34.24 min cyanuric acid, 41.57 min 2,4,7-trihydroxypteridine. In this case the GC-MS method used was the method A described in [28].

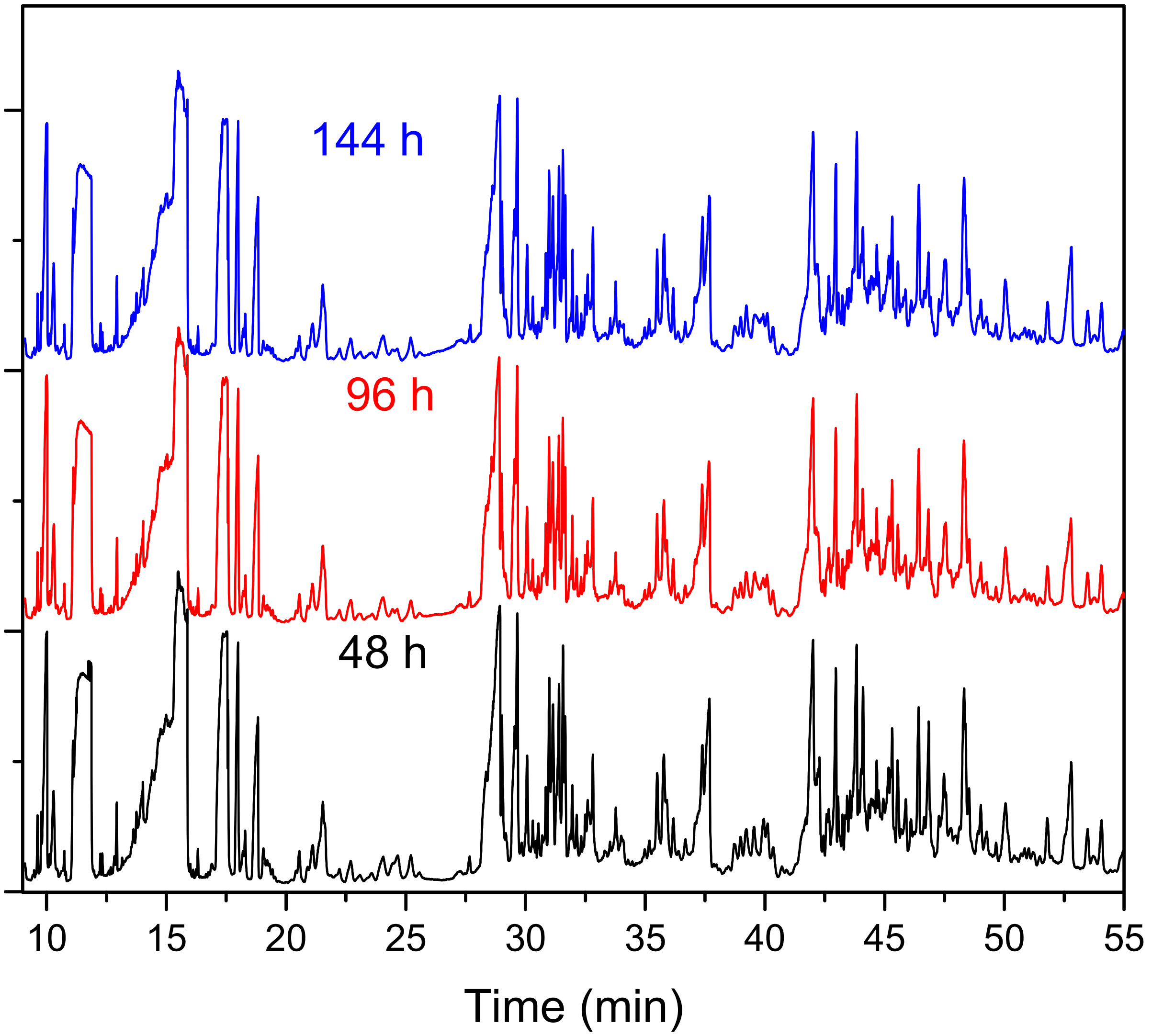

Figure S8. Representative GC-MS chromatograms of acid hydrolysed insoluble NH_4CN polymers, gel fractions, synthetized at 80 ºC, ref. [10] in the main manuscript. The time showed over each chromatogram is the polymerization time. The polar organic compounds identified in these samples were (the retention time for each analyte is indicated): 9.62 min lactic acid, 9.86 min glycolic acid, 12.93 min malonic acid, centered at 15.50 min urea+glycine, 16.32 min uracil (co-elution peak with pyrazine-2,5-diol), 18.20 min hydroxymalonic acid, 19.05 min aspartic acid, 21.10 min 2,2-dihydroxymalonic acid, 21.54 min aminomalonic acid, 24.06 min 5-hydroxyhydantoin, 26.67 min aspartic acid, 28.91 min ammelide + 2-amino-4-hydroxyimidazol (co-elution peak of several analytes), 29.02 min barbituric acid, 29.66 min cyanuric acid (tentatively, co-elution peak), 30.70 min tartaric acid, 31.40 min 5-aminouracil + 2-amino-4,6-dihydroxypyrimidine (co-elution peak), 32.81 min orotic acid, 35.78 min adenine, 36.17 min citrazinic acid (co-elution peak), 37.70 min uracil-5-carboxylic acid (co-elution peak), 40.71 min 8-hydroxyadenine, 41.60 min 2,4,7-trihydroxypteridine, 43.84 min guanine, 44.67 min isoxanthopterine, 46.43 min leucopterine, 46.83 min 2,6-diaminopurine. In this case the GC-MS method used was the method A described in [28].

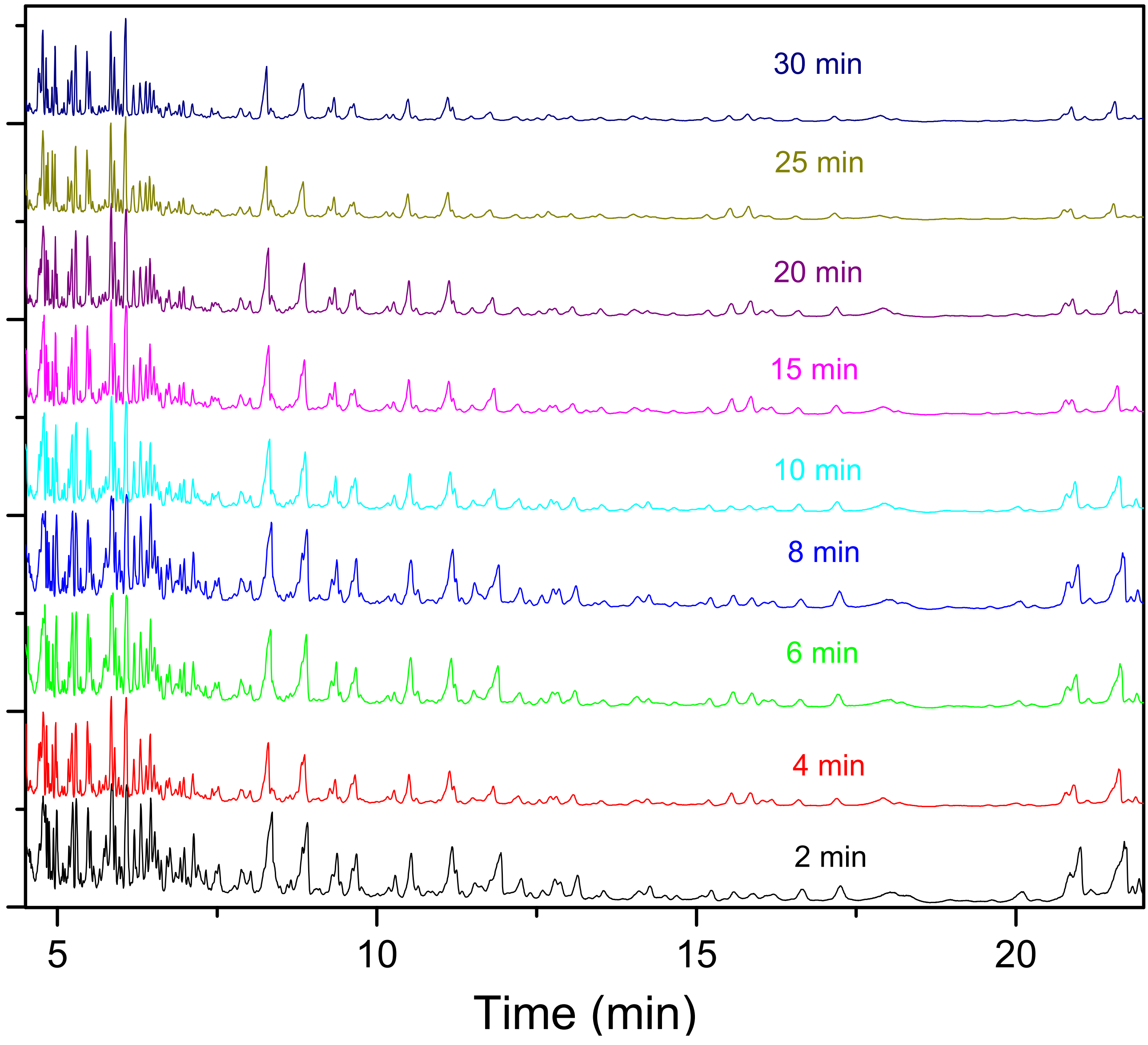

Figure S9. Representative GC-MS chromatograms of acid hydrolysed insoluble NH_4CN polymers, gel fractions, synthetized under hydrothermal conditions at 180ºC using a N_2 atmosphere. The time showed over chromatogram is the polymerization time. The polar organic compounds identified in these samples were (the retention time for each analyte is indicated): 4.47 min glycine (3 TMS, co-elution peak), 4.59 min fumaric acid, 4.78 min uracil + 2,5-dihydroxypirazine (co-elution peak), 4.82 min hydroxymalonic acid, 4.98 min malonic acid, 5.16 min dihydroxymalonic acid, 5.22 min aminomalonic acid, 5.28 min malonic acid, 5.35 min adipic acid, 5.46 min aspartic acid, 5.90 min barbituric acid, 5.94 cyanuric acid, 6.29 min threonine, 6.38 min 5-aminouracil, 6.48 min + 6.69 min isomers of 2-amino-4,6-dihydroxypyrimidine (NIST), 6.97 min orotic acid, 8.34 min adenine, 9.23 min citrazinic acid, 10.48 min 2,4,7-trihydroxypteridine, 10.94 min isoxanthopterine, 12.51 min guanine, 8-hydroxyadenine, 17.17 min leucopterine, 18.89 min 2,4,6,7-tetrahydroxypteridine, 20.75 min 7-amino-isoxanthopterine (tentatively assigned by its mass spectra *m/z* 482, 467). In this case the GC-MS method used was the method B described in [29].
